# Natural-Based Biomaterial for Skin Wound Healing (Gelatin vs. Collagen): Expert Review

**DOI:** 10.3390/polym13142319

**Published:** 2021-07-14

**Authors:** Ruth Naomi, Hasnah Bahari, Pauzi Muhd Ridzuan, Fezah Othman

**Affiliations:** 1 Department of Human Anatomy, Universiti Putra Malaysia, Serdang 43400, Selangor, Malaysia; ruthmanuel2104@gmail.com (R.N.); haba@upm.edu.my (H.B.); 2 Estika Research Centre, Kuala Terengganu 21080, Terengganu, Malaysia; drpmridzuan@gmail.com; 3 Department of Biomedical Sciences, Faculty of Medicine and Health Sciences, Universiti Putra Malaysia, Serdang 43400, Selangor, Malaysia

**Keywords:** collagen, gelatin, wound healing, in vitro and in vivo, biomaterials scaffolds, physicochemical property

## Abstract

Collagen (Col) and gelatin are most extensively used in various fields, particularly in pharmaceuticals and therapeutics. Numerous researchers have proven that they are highly biocompatible to human tissues, exhibit low antigenicity and are easy to degrade. Despite their different sources both Col and gelatin have almost the same effects when it comes to wound healing mechanisms. Considering this, the bioactivity and biological effects of both Col and gelatin have been, and are being, constantly investigated through in vitro and in vivo assays to obtain maximum outcomes in the future. With regard to their proven nutritional values as sources of protein, Col and gelatin products exert various possible biological activities on cells in the extracellular matrix (ECM). In addition, a vast number of novel Col and gelatin applications have been discovered. This review compared Col and gelatin in terms of their structures, sources of derivatives, physicochemical properties, results of in vitro and in vivo studies, their roles in wound healing and the current challenges in wound healing. Thus, this review provides the current insights and the latest discoveries on both Col and gelatin in their wound healing mechanisms.

## 1. Biomaterials

Biomaterials are substitutes for biological tissues in the human body that have the ability to interact with the body. They can either be natural or synthetic. For more than 50 years, biomaterials have been developed to be used in the therapeutic field [1]. Approximately, the annual growth rate for biomaterials in the global market is 15.97 % and by the year 2027 it is expected to hit up to USD 348.4 billion in value [2]. Being natural biomaterials, both collagen (Col) and gelatin have shown promising results for skin wound healing. With regard to this statement, Col and gelatin are highly biocompatible toward human tissues as they resemble the extracellular matrix [3,4]. This property makes both of these materials the main choices for implantable medical products for in vitro testing [5]. Gelatin is the hydrolysed form of Col and both of them have the same amino acids, but their chemical properties differ.

### 1.1. Collagen

Col is a fibrous protein consisting of more than 1000 amino acids and is a substitute for cell scaffolding in the human body. It is essential for cell signaling, resilience for multicellular organisms and resistance to mechanical stress. In humans, Col is the most predominant protein as it makes up the major component of the extracellular matrix (ECM) [6]. Col can either exist as a homotrimer or a heterotrimer, depending on the composition of the α-chains. To date, there are 29 types of Col which have been identified. Among them, Col type I (Col-I) is the most abundant form widely incorporated into therapeutics, due to its high biocompatibility and low immunogenicity [7]. It makes up 90% of the human skin composition [8] and 30% of bodily proteins [6]. The structure of Col appears in a triple-helical domain, self-twisted into a rope-like structure. It consists of repeating triplets of Gly-X-Y, with glycine being constant, while x and y can be either proline or hydroxyproline. This structure is held together by hydrogen bonds which ensure its stability. This bond is highly resistant to cleavage which can only be disrupted by the presence of collagenase [8]. Such characteristics further contribute to Col being widely used in the medical field. However, Col can still induce a very low amount of antigenicity. However, this can be overcome through crosslinking the Col by removing the telopeptide molecules [9]. Naturally, the binding of Col with glycoprotein prevents the rise of an immune response toward Col in the body. Col has the ability to control the cycle of cell migration, proliferation, and differentiation through the action of fibroblasts. In the preparation of a low molecular weight of Col, the high cost, presence of impurities, enzymatic degradation, poor elasticity and degree of crosslinking are great challenges which could hinder its incorporation in certain fields [10].

### 1.2. Gelatin

Gelatin is a natural polymer that can be derived from nonsoluble Col through hydrolysis [11]. Since gelatin is a Col derivative, it possesses almost the same characteristics as Col. Gelatin is proven to be biocompatible to human tissues, flexible, stable and can be modified to act as a scaffold base [12]. Gelatin comprises of proline, glycine and hydroxyproline [11] and is similar in the composition of amino acids as Col, mimicking the extracellular matrix [13]. The composition of glycine amino acid in the gelatin is responsible for the adherence of cells [14]. The structure of gelatin mainly depends on the extraction process. However, it consists of randomized macromolecular and heterogeneous structures with a poor melting point. [11]. Human tissue has the ability to metabolise gelatin and this is the main reason for gelatin becoming one of the choices in the pharmaceutical industry. Its function can always be customized and it does not trigger any immune response in the human body [15]. Due to these unique characteristics, gelatin is in high demand in the global market, which was approximately 412.7 kilotons in 2015, with pigskin being the primary source of gelatin extraction [16]. Based on the process of extraction, gelatin can be categorized into two types, namely, type A and type B. Type A (positively charged) refers to acid extraction, while type B (negatively charged) refers to alkaline extraction [17,18]. During extraction, the generated isoelectric point and variation enhance the binding of the gelatin to the charged therapeutic agents [19]. The isoelectric point for type A and B gelatins are usually at pH 9 and pH 5, respectively, [14]. Gelatin can be obtained from a very low cost commercially available source and possesses a high range of biodegradability [20]. The drying temperature of gelatin influences its properties and it possesses a perfect film-forming property. This makes gelatin suitable to be widely used as a drug delivery substance. Moreover, gelatin is known to be a barrier against gas and hydrophilicity, although it exhibits poor mechanical strength [21]. However, the rapid degradation of gelatin in a colloidal solution and at 37˚C still remains a challenge in biomedical applications [22]. However, the adverse effects of gelatins can be influenced by crosslinking agents [16].

## 2. Differences between Collagen and Gelatin

Both Col and gelatin are natural biomaterials that are being widely used as therapeutics, particularly in skin wound healing. However, some differences exist which make one superior to the other. The differences are presented in Table 1 [10,23,24,25,26,27,28,29,30].

### 2.1. Sources of Collagen and Gelatin

There is a vast range of sources that could be used to extract Col and gelatin. Although the source of derivatives influences the outcome, it still can be modified according to the medical needs. Col is commercially available and can be extracted literally from any sources, even from 75-million-year-old preserved dinosaur tissues [28]. Similarly, since gelatin is a Col derivative, the process of obtaining and the sources of gelatin are also wide. The sources of Col and gelatin are summarized in Table 2 [16,29,30,31,32,33,34,35,36,37,38,39,40,41,42].

### 2.2. Physicochemical Properties of Collagen and Gelatin for Skin Wound Healing

Collagen and gelatin exhibit properties that are suitable to accelerate skin wound healing. Although both are approved natural biomaterials for skin wound healing, yet their biological properties greatly differ from one another. Yet, both can be ideal biomaterials for skin wound healing. The properties of Col and gelatin are discussed below.

#### 2.2.1. Thermal Resistance

Thermal resistance indicates the ability of the scaffold to be resistant to heat. This is essential to ensure that the elasticity, toughness and strength of the scaffold is maintained upon changes in the temperature. The functionality of the biomaterial must be preserved at the wound site. The transition temperature for acid-soluble Col (115.33 ± 0.97 °C) and pepsin-soluble Col (112.1 ± 0.79 °C) is highest at a heating rate of 2 °C min^−1^. The maximum transition temperature was recorded at 39.6 and 110.7 °C for acid-soluble Col, while for pepsin-soluble Col, it was at 38.33 and 109 °C. At the same time, heating for 0.5 min^−1^ was recorded at 39.6 and 38.33 °C for acid-soluble Col and pepsin-soluble Col, respectively [43]. In contrast, gelatin designed in the form of a gel was proven to be completely thermo reversible. The melting point for gelatin was recorded at 16 °C, while the gelling temperature was at 5 °C [44].

#### 2.2.2. Chemical Stability

The chemical stability of any biomaterial scaffold is essential for its functional stability and this is usually confirmed through Fourier transform infrared spectroscopy (FTIR). A uniformity in the major absorption band indicates scaffold stability [45]. FTIR for Col was recorded at 1632 cm^−1^, 1548 cm^−1^ and 1237 cm^−1^ for amide I, amide II and amide III, respectively. The major absorption band was recorded at 2923 cm^−1^, indicating uniform CH_2_ stretching [46]. As for gelatin, the major absorption bands were seen at 3433 cm^−1^, 1630 cm^−1^, 1565 cm^−1^ and 1240 cm^−1^ for amide A, amide I, amide II and amide III, respectively. The recorded major absorption band for gelatin ranged from 1460 cm^−1^ to 1380 cm^−1^, which highly corresponded to the presence of the methyl group [32].

#### 2.2.3. Mechanical Strength

An ideal scaffold must be able to withstand force, particularly a scaffold that has been designated for wound healing. In this scenario, mechanical strength determines whether a scaffold is able to withstand the force or not [47]. Ghodbane et al., (2016) stated that the ultimate stress values of ovine, bovine, and porcine were recorded at 15.08 kPa ±15.08 kPa, 12.33 kPa ± 2.37 kPa and 13.91 kPa ± 3.11 kPa, respectively. In addition, the strain percentage and tensile toughness for Col were 50.74% ± 4.02% and 3.32 kJ/m^3^ ± 0.81 kJ/m^3^ [48]. A study done by Xing et al., (2014) [49] described that unpurified gelatin exhibited better mechanical strength in comparison to purified gelatin. As reported by them, the average storage and loss of modulus of the gelatin were 2.83 ± 0.10 fold and 3.80 ± 0.43 fold, while the ultimate stress was recorded as 107 kPa ± 25 kPa [49].

#### 2.2.4. Oxygen Barrier

An ideal scaffold must be greatly permeable to allow the diffusion of oxygen to enhance the wound healing mechanism [50]. An adequate pore size of the scaffold determines this property of the scaffold. For adult skin healing, the optimal pore size has been determined to be in the range of 20 μm to 125 μm [51]. As stated by Ghodbane et al., (2016) [48], Col derived from ovine, bovine and porcine sources exhibited average mean pore sizes of 73.05 µm ± 10.79 µm, 85.84 µm ± 9.51 µm and 87.32 µm ± 10.69 µm, respectively. All Col scaffolds display heterogenous architectures, despite the sources of the derivative [48], while gelatin scaffolds display open pore structures, highly interconnected and widely tuneable. In addition, the pore size of gelatin can be altered according to the specific need by using ice particulates without affecting its mechanical property [52].

#### 2.2.5. Water Vapor Transmission Rate (WVTR)

The WVTR directly corresponds to the ability of the scaffold to retain moisture in accordance with the need of the skin-healing phase. In relation to this, 2028.3 ± 237.8 g/m^2^/day has proven to be an ideal value to retain the moisture environment at the injury site to promote healing [53]. Col shows a WVTR of s 4750 ± 700.209 g/m^2^/day [54] while for gelatin it is 1040 ± 95 g/m^2^/day [55].

#### 2.2.6. Biocompatibility

The main purpose of designing a scaffold is to promote the migration and proliferation of the cells toward the scaffold in order to promote healing. This can only be achieved if the designed scaffold is compatible to human cells. Mousavi et al., (2019) described that Col enhanced the binding of cells toward the scaffold through integrin binding and the presence of a cell-producing enzyme which was capable of breaking it down directly. This is possible due to the existence of the amino acid sequence in the backbone of Col, arginine-glycine-aspartic acid, which is similar to that in the human body. As a result, cell adhesion and proliferation are accelerated [54]. Meanwhile, gelatin scaffolds have been proven to have no significant effect on cell proliferation and cytotoxicity against human adipose-derived mesenchymal stem cells (hASCs. In view of this, gelatin is also categorized as a compatible biomaterial for human cells [56].

#### 2.2.7. Biodegradability

To ensure a proper remodelling process, the scaffold must be able to degrade in an appropriate time. Since serine protease and collagenase can degrade Col naturally through intracellular degradation, it is thus controlled locally by engineered tissue cells. At the same time, metalloproteinases (MMPs) secreted by the inflammatory cells in the human body can degrade the Col (extracellular degradation) [57]. Meanwhile, gelatin degradation duration ranges from 35 days to 63 days in the presence of lysine diisocyanate ethyl ester [58].

#### 2.2.8. Immune Response

The stimulation of an immune response is a very relevant issue when it comes to a biomaterial. An ideal biomaterial should not trigger any immune reaction by the host tissue. In relation to this, Col induces a very small amount of immune reaction when a scaffold is newly being placed onto the human skin. This happens depending on the source of the extracted Col, crosslinking agent or the presence of remnants of cells [58]. However, this can be overcome by removing the terminal telopeptide from the Col molecule. Upon the removal of that particular structure, the arrangement of the Col fibril pattern is disrupted, leading to an amorphous structure. When this happens, the surface of Col becomes highly soluble due to the positive electrons, eliminating the immunogenicity property of the Col scaffold [59]. Meanwhile, gelatin is proven to stimulate immunogenicity in the host tissue too. However, this is two-fold lower compared to Col. Specifically, when gelatin is crosslinked, a covalent bond is formed. This in turn creates a second network locked in place by chemical bonds [60]. It is widely speculated that biomaterials must be covalently attached to a bigger protein in order to elicit an immune response [61]. A covalent bond can become securely bound to a carrier molecule, most commonly a protein, if it exists. The hapten-carrier complex induces antibody formation, and so becomes immunogenic. The hapten then reacts with the antibodies that have been produced against it, causing an immunological or allergic response [62]. Introducing a covalent net point to gelatin manifested in it suppressing its immunogenicity property [58].

#### 2.2.9. Antibacterial Property

Integrating an antibacterial effect into a biomaterial is an essential component in preventing the entrance and colonisation by microbes at the injury site. Native Col does not possess antibacterial properties. Hence, the incorporation of an antibacterial effect in Col further contributes to its physicochemical characteristics. Col can be integrated with silver nanoparticles (AgNP) [63], collagencin [64], epoxidized safrole [65], polyhexamethylene biguanide [66], titanium dioxide [67], etc. Similarly, native gelatin does not show any antibacterial effect. However, to give it such an effect, gelatin can be easily integrated with Ginkgo biloba extract [68], D-Limonene [69], Finibax [70], black pepper oleoresin [71], etc.

#### 2.2.10. Hemostatic Effect

Hemostasis (stoppage of bleeding) is the primary component in wound healing. In this, native Col plays a vital role as a hemostatic agent as it serves as a major activator for immediate platelet response when there is injury. Furthermore, it allows the platelets to adhere at the wound site, forming a clump or clot together to stop the bleeding [72]. At the same time, this property can be further improved by integrating Col scaffold with oxidized microcrystalline cellulose [73]. On the other hand, gelatin does stimulate the hemostatic process by hastening the development of thrombus and providing it with a structural support. Within two days and up to six weeks, gelatin scaffold has proven to be absorbed or liquefies upon being utilized as a hemostatic agent. In comparison to Col, gelatin has been shown to be a superior hemostatic agent [74].

#### 2.2.11. Cytotoxicity

Cytotoxic is an important parameter to be considered when it comes to a biomaterial. It determines whether the scaffold supports cell viability or not. Col has been proven to not induce cytotoxic effects, particularly in human cells. Instead, Col aids in cell delivery and distribution by creating a microenvironment for the uniform spreading and proliferation of cells [75]. Similarly, native gelatin does not exhibit any form of cytotoxic effect upon being seeded with keratinocytes. However, the method of gelatin extraction can trigger cytotoxic effects in the gelatin scaffold. For instance, >0.5 mg/mL enzymatic extraction could trigger the cytotoxic effect [76].

#### 2.2.12. Cell Proliferation

Cell growth is an essential element in wound healing. With regard to this, the native col scaffold has been proven effective for cell attachment and proliferation, particularly at the wound site. Interestingly, the cells at the wound site appeared to be symmetrical and aligned in accordance with the native Col scaffold [77]. Nonetheless, an in vitro study proved that col scaffolds successfully accelerated healing by enhancing proliferation of fibroblasts [78] by increasing biological and structural integrities which resembled the native extracellular matrix (ECM) at the wound site [79]. In contrast, gelatin has been classified as a biofriendly scaffold which interacts perfectly with adipocytes, keratinocytes, cerebellum stem cells, and pre-osteoblasts, due to its similar structure to ECM. So the gelatin scaffold is able to enhance the vascularization process within the newly engineered tissues at the healing site [56].

## 3. Roles of Collagen and Gelatin in Wound Healing Phases

Col makes up the major component of the extracellular matrix (ECM) in humans [4] while gelatin is a Col derivative [12]. Col is a perfectly organized structure as a three-dimensional (3D) scaffold that surrounds the cells. Thus, it has a dominant influence in maintaining the structural and biological integrity of the ECM. Being a natural component of the human body, it has been categorized as one of the major biomaterials widely used in wound healing [4]. Nonetheless, the physicochemical properties of Col and gelatin greatly influence the mechanism of skin wound healing.

Col and gelatin can penetrate into lipid free interferences (membranes) and are surface-active molecules [80,81]. In addition, they are thermally and chemically stable with high tensile strength, permeable to O_2,_ highly biocompatible, regardless of the source of the derivative, biodegradable, weakly antigenic and hemostatic agents. They have the ability to form high tensile, stabilized fibers through self-aggregation and crosslinking. These fibers can be modified into any form of scaffolding [63]. Particularly in skin injury, Col primarily, acts as a chemotactic agent by creating a microenvironment for the initiation of the healing mechanism (inflammatory phase) as it forms a protective barrier for the skin [82]. Similarly, gelatin is able to act as a hemostatic agent to initiate the wound healing mechanism and to absorb exudates present at the wound region while creating a suitable microenvironment for the inflammatory phase to take place [83].

In the proliferative phase, the wound bed prepares for the growth of new tissues and the wound undergoes contraction. In this phase, Col aids the growth of new tissues and accelerates the deposition of granulation tissues [84]. At the same time, Col enhances the activity of fibroblasts, leading to a drastic increase in the fibroblast proliferation rate. By means of contraction, Col forms a network that reinforces the adhesion of cells and tissue integrity [85]. As for gelatin, it acts as a porous scaffold to stimulate the migration of cells, specifically fibroblasts to the injury site. It further enhances the formation of new tissues by providing structural and mechanical strength at the wound site [83]. In the maturation phase, the continuity of the skin begins and the process of re-epithelisation continues [84]. In this process, Col supplies adequate nutrition directly to the wound, enhancing the repair mechanism and aiding in scar reduction [86].

## 4. Collagen and Gelatin for Skin Wound Healing

To date there is numerous scientific research on the effectiveness of including Col and gelatin in skin wound healing. Being biocompatible, they are beneficial and superior compared to other available natural products. Col is naturally found in the human body while gelatin is a hydrolysed form of Col. Studies done by Dill and Morgelin (2020) [77] and Wiegand et al., (2016) [87] showed that native Col provided a 3D microenvironment that stimulated cell proliferation and aided the migration of keratinocytes and fibroblasts to the physiologic locations during wound healing. Through their in vitro investigations, they noticed that fibroblasts and keratinocytes attached to Col with high affinities. Similarly, Jridi et al., (2015) [88] observed that positive interaction existed between Col and human cells, leading to an increased level of hydroxyproline at the injury site. With regard to this, in vivo experiments carried out by Dang et al., (2015) [89], Chen et al., (2019) [90], Ke et al., (2015) [91], and Helary et al., (2015) [92] further proved that Col had the capacity to increase cellularity, granulation tissues, expressions of EGF, FGF, and CD31, collagenization, neovascularization and re-epithelization. This thereby resulted in rapid healing at the injury site. Interestingly, there was no immune response observed, despite the varying sources of the Col derivatives used in the in vivo testing. The outcomes clearly indicated that Col derivatives of any source were highly biocompatible to humans, due to their integrin (RGD) components [10]. Table 3 shows the in vitro and in vivo evidence of native collagen for skin wound healing.

Similarly, native gelatin showed good adhesion to fibroblasts and cell viability from 72 h up to 7 days [93,94]. The in vivo studies done by Hsu et al., (2019) [95], Nikpasand et al., (2019) [96], and Jang et al., (2017) [97] indicated the formation of thick granulation tissues, increased re-epithelization and blood vessel formation with an absence of cytotoxic effects confirmed through CCK-8 assays. For both Col and gelatin scaffolds, the analysed physicochemical properties correlated with those of an ideal scaffold for wound healing as per discussed in the physicochemical section above. No contraindication outcome was observed in the usage of Col and gelatin for skin wound healing. Table 4 shows the in vitro and in vivo evidence of native gelatin for skin wound healing.

**Table 3:** In vitro and in vivo studies of native collagen for skin wound healing.

Author	Objective	Study Design	Subject	Duration	Outcome	Conclusion
Dill et al., (2020) [77]	To access the efficacy of native Col template in wound healing	In vitro	Keratinocytes and fibroblasts cells	15 min to 180 min	‒ Large amount of filopodia found arise from the cell body and attach to the Col scaffold. ‒ Attachment of keratinocytes and fibroblasts to the scaffold increased drastically over time. ‒ >30 min, maximum spreading of cells with well-defined morphology was seen.	Col scaffold able to stimulate cell proliferation and aids the migration of keratinocytes and fibroblasts to the physiologic locations during wound healing.
Wiegand et al., (2016) [87]	To evaluate the effectiveness of native Col matrix (NCM) in wound healing	In vitro	NIH-3T3 fibroblasts cells	1 h to 14 days	‒ Atomic force microscopy revealed that NCM fibril similar to human dermis’s microstructure. ‒ NCM exhibit open, single porous structure under scanning electron microscopy (SEM). ‒ Drastic increase in fibroblast was observed by day 14. ‒ NCM binds with matrix metalloproteinases-2 (MMP-2) with high affinity. ‒ Stabilization of growth factor (GF) was seen.	Col provides a 3D microenvironment that promote ingrowth of fibroblasts, migration and cell proliferation
Jridi et al., (2015) [88]	To evaluate the efficacy of Col gel in wound healing	In vivo	18 female Wistar rats	Up to 12 days	‒ SEM showed thick dense fiber with low porosity. ‒ High interaction between the triple helical structure. ‒ Increased level of hydroxyproline at the injury site. ‒ Absence of inflammatory cells. ‒ Dense formation of neovascularization and connective tissue was seen. ‒ Rapid wound closure was seen on 8th day. ‒ Drastic decrease in exudation and swelling was noted. ‒ Increase rate of re-epithelization and wound contraction was seen.	Col can rebalance the environment in chronic wound, thereby enhancing rapid wound healing.
Dang et al., (2015) [89]	To access the efficacy of Col from haddock skin for wound healing	In vivo	18 male Balb/c mice	Up to 22 days	‒ UV absorbance spectrum indicated the presence of polypeptide chains and aromatic amino acids. ‒ Absorbance of FTIR showed the existence of stronger hydrogen bond. ‒ Denaturation temperature recorded at 24.9 °C and endothermic peak at 47.6 °C. ‒ Decrease in bleeding and clotting time was seen. ‒ Increase in cellularity, quantity and maturity of epidermal layer and blood vessels was seen.	Col shows positive outcome for scalded skin healing
Chen et al., (2019) [90]	To access the efficacy of fish Col in wound healing	In vivo	63 female SD rats	3, 7 and day 14	‒ Increased level of hydroxyproline in the treatment group. ‒ Granulation tissue formation and fibroblasts increased in Col group. ‒ Reduction in inflammation and increase in re-epithelization was seen. ‒ Expression of EGF, FGF, and CD31 was seen in the treated group. ‒ Complete wound closure was seen in day 14.	Regardless of method of extraction, Col accelerates wound healing process.
Ke et al., (2015) [91]	To access the effectiveness of Col sponge integrated with skin-derived precursors for skin wound healing	In vivo	Male C57BL/6J mice	Up to 14 days	‒ The cell density increased over time up to 14 days. ‒ Drastic reduction in residual area was noticed. ‒ Complete wound closure recorded on day 7. ‒ Formation of fibrin crust was seen on day 14. ‒ Thicker layer of epidermal and expression of isolectin and vWF was observed in experimental group.	Col sponge integrated with skin-derived precursors accelerates healing mechanism through paracrine secretion.
Helary et al., (2015) [92]	To study the effectiveness of dense Col matrix for chronic wound healing	In vivo	12 adult Wistar male rats	15 and day 30	‒ Elastic modulus of the scaffold ranges from 1 to 10 kPa. ‒ Homogenous fibrillar network (<50 nm) and high porosity was recorded. ‒ Constant increase in the swelling ratio was observed up to day 4. ‒ Scaffold loaded with ampicillin exhibited antibacterial effect up to day 4 with a minimum inhibitory concentration of 250 ng mL^−1^. ‒ Fibroblast cells viable up to 24 h. ‒ Fibrous cap, absence of inflammation and CD68 was recorded in the in vivo model.	Col accelerates the healing mechanism in chronic wound model.
Wang et al., (2020) [98]	To study the effect of dermal Col matrix in full thickness skin wound	In vitro and in vivo	L929 fibroblast cells, 3 rabbits, 20 mice and 40 rats	Up to 90 days	‒ Absence of pyrogenic effect in rabbits. ‒ Cell attachment was seen from 12 h onwards. ‒ Proliferation of cells visible from 48 h. ‒ Complete degradation of the matrix was recorded on the 90th day. ‒ Decrease in the wounded area and wound closure was seen from 2nd week onwards. ‒ Collagenization, re-epithelization and attenuated inflammation was seen in the treated groups.	Col matrix can serve as a dermal substitute and is able to regenerate full thickness skin loss.
Akturk et al., (2016) [99]	To access the efficiency of Col in skin wound healing	In vitro and in vivo	3T3 fibroblasts, HaCat keratinocytes and 40 male Wistar albino rats	Up day 14	‒ For Col matrix the FTIR absorption band was recorded at 1600 cm^−^1 to 1700 cm^−1^, 1500 cm^−1^ to 1550 cm^−1^, 1200 cm^−1^ to 1300 cm^−1^. ‒ The pore size of the scaffold ranged from 10 µm to 200 µm with tubular channel. ‒ Complete degradation of scaffold was recorded on day 7. ‒ The tensile strength, elongation at break and elastic modulus of the scaffold recorded at 0.099 MPa to 0.022 MPa, 15.43 % to 2.38 % and 0.73 MPa to 0.16 MPa respectively. ‒ Absence of cytotoxic effects. ‒ Proliferation of cells was seen from 3rd day of incubation with spindle-like shape and stretched pseudopod cells spreading. ‒ Re-epithelization, neovascularization and granulation tissue formation was seen. ‒ Complete wound closure was achieved by 14th day.	Col aids skin wound healing with absence of cytotoxic effect.
Zhou et al., (2016) [100]	To study the outcome of tilapia skin derived Col sponge in wound healing	In vitro and In vivo	Immortalized human keratinocytes (HaCaT) and 8 male Sprague Dawley rats	Up to 28 days	‒ The tensile strength of Col nanofiber was ideal 6.72 ± 0.44 MPa. ‒ The contact angle was recorded at 26.71 ± 4.88°. ‒ Absence of immune response for IgG and IgM. ‒ Thermal stability and swelling capacity was recorded. ‒ FTIR indicated the maintenance of α-helical structure after crosslinking. ‒ Rate of cell proliferation (HaCaT) reached 114% on 5th day. ‒ Expression of TGase1, filaggrin and involucrin gene was seen. ‒ Complete wound closure was seen on 14th day.	Electro-spun tilapia skin derived Col enhance rapid wound healing.
Busra et al., (2019) [41]	To study the effectiveness of ovine-derived Col for full thickness healing	In vitro and in vivo	human epidermal keratinocytes (HDF), human dermal fibroblasts (HDF) and 30 athymic/nude mice	Up to 13 days	‒ Heterogenic porous structure was seen on the Col scaffold. ‒ Absorbance band of FTIR correlated with Col-I property; 3302 cm^−1^ (amide A), 2927 cm^−1^ (amide B), 1632 cm^−1^ (amide I), 1548 cm^−1^ (amide II) and 1237 cm^−1^ (amide III). ‒ Absence of cytotoxic effect and immune response. ‒ At 13th day wound closure was seen with absence of crust. ‒ 4 to 5 cell layers of thick proliferative basal keratinocytes and tight junction were seen in the experimental group.	Ovine tendon derived Col; with or without crosslinkers enhance wound healing
Pal et al., (2016) [101]	To study the outcome of Mrigal fish derived Col in full thickness wound healing	In vitro and vivo	Fibroblasts/ 24 adult Wistar rats	3, 5, 7, 10 and day 15	‒ High fraction of Col-I with D-spacing was seen. ‒ The FTIR showed an absorption band of 3323 cm^−1^ (amide A), 2938 cm^−1^ (amide B), 1661 cm^−1^ (amide I), 1548 cm^−1^ (amide II) and 1237 cm^−1^ (amide III). ‒ The denaturation temperature of scaffold recorded at 32 °C. ‒ Interconnected high porous structure with a swelling capacity ~ 410 % was seen. ‒ Elongation and proliferation of fibroblasts was seen from day 3. ‒ Expression of keratin 14, keratin 10 and E-cadherin was detected. ‒ Increased expression of TGF-β1 was seen from 3^rd^ day. ‒ By day 15, >98% wound closure was seen. ‒ Rapid migration and distribution of Col was seen. ‒ Experimental group showed presence of skin appendages by day 10.	Col can increase the rate of wound healing, dermal reconstitution and re-epithelization.
Masry et al., (2018) [102]	To access the efficacy of stabilized Col matrix (SCM) for wound healing	In vitro and In vivo	HaCaT and male C57BL/6 mice	Up to day 14	‒ The stiffness of the SCM ranged from 1 to 5 MPa. ‒ Infiltration of cells seen on day 3 which gradually resolved on day 7. ‒ Increased level of efferocytosis index was observed in the experimental group. ‒ Increase in IL-1b, TNF-a, IL-10 and VEGF was recorded. ‒ Inhibition of biofilm, wound closure by day 14 and presence of high degree of Col deposition was seen in the SCM treated group.	Stabilized Col matrix increase rate and quality of healing.

## 5. Collagen as a Drug Carrier in the Pharmaceutical Industry

In the pharmaceutical field, Col modified into nanospheres, nanoparticles or microspheres is used as a device for drug delivery in wound healing which may be formulated either through emulsification, desolvation, coacervation, spray drying, milling technique, fluidization, solvent precipitation method, extrusion, or interfacial polymerization [107]. In this form, they have been proven to be effective in penetrating into the systemic circulation with the aid of Col. This is made possible as the integration of Col has resulted in a larger wound surface area covered due to its small size, high absorption ability and capacity to form a colloidal solution [108]. Apart from this, the crystallite suspension in the gel aggregates emerges as a multiplex chain system in the Col fold configuration. This characteristic eases the formulation of aggregates into colloidal drug delivery carriers [109]. Meanwhile, the formation of nanospheres is regulated by a blend of different electronic and electrostatic forces, with sodium sulphate acting as the liquefying reagent to allow for maximum charge to charge linkages between DNA plasmid and Col, whereas for Col nanoparticles the molecular weight of the Col influences the stability of the Col nanoparticles [109]. Nonetheless, changes in pH and temperature have proven to have significant impact on the molecular weight of the collagen solution and the noncovalent linkage responsible for the collagen’s molecular structure during the formation of Col nanoparticles [110].

**Figure 1:**
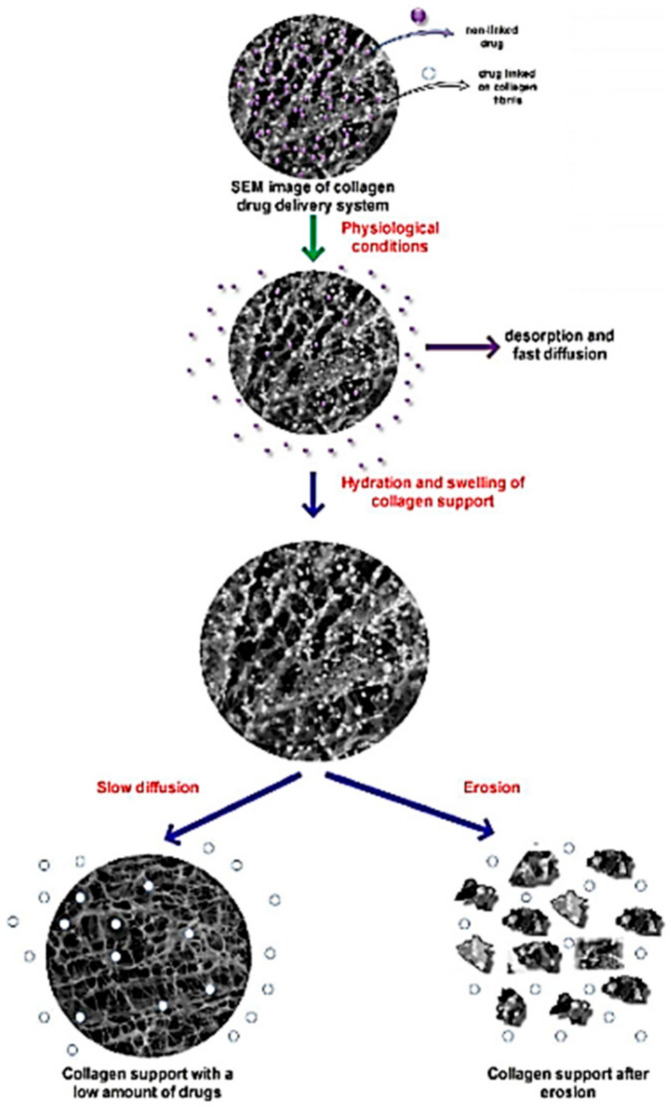
Mechanism of collagen matrix as drug carriers as used under the creative commons attribution 3.0 license [112].

Col nanoparticle and nanosphere drug delivery carriers are easy to sterilise and, at the same time, they facilitate and accelerate the uptake of exogenous substances. For instance, the uptake of anti-HIV in a variety of cells, specifically from macrophages in antiretroviral therapy in nanotechnology, has always been easy with a Col biomaterial, in comparison to other drug carriers [111]. In addition, Col nanoparticles enhance the delivery of certain drugs such as camptothocin, tetracycline, doxycicline, rolitetracycline, minocycline, amikacin, tobramycin, vancomycin, etc., into the systemic circulation due to its penetration ability into colloidal solution [112]. Conversely, the kinetics in a Col biomaterial are usually influenced by the physical processes, such as swelling to form a gel, polymer hydration via fluid, drug diffusion through the gel formed and eventual erosion of the polymeric gel [113]. For example, the polymer undergoes a relaxation process in an aqueous media, resulting in direct, gradual erodation of the hydrated polymer. It is probable that, like the sponges, it swells and dissolves at the same time, with each of these processes contributing to the overall release mechanism. The diffusion rate of the medium represented by biological fluid in the polymeric sponge, on the other hand, determines the amount of drug released [114]. The main cause of kinetics in this condition is polymeric sponge erosion after water diffusion, as well as the swelling ratio. Aside from that, the drug release kinetics of Col can be affected by various chemical crosslinkings that affect the degradation rate, such as changes in porosity, density, and other factors [115]. Figure 1 shows the kinetics of drug release from the Col matrix [112]. Table 5 shows the current FDA approved skin healing products from native collagen.

## 6. Gelatin as a Drug Carrier in Wound Healing

Gelatin, being a Col derivative, is highly biocompatible to be inculcated as a drug delivery wound dressing. Specifically, gelatin-based antibacterial wound dressing produced via casting has been proven as an effective dressing material. The outcome indicates it has hydrolytically and thermally stable properties, increased mechanical strength, and exhibits the optimum range of hydrophilicity as well as porosity, which accelerate the healing mechanism [116]. In addition, gelatin crosslinked with diosgenin-conjugated nanocellulose loaded with neomycin shows protection against S. aureus and E.coli in a dose dependent manner. Similarly, the pH of the biomaterial does influence the release of drugs in this situation. For instance, 60% of neomycin was released from the gelatin at pH 5.5 while 40% was released at pH 7.4 within 15 min [117]. Nevertheless, another study shows that combining gelatin with poly(ε-caprolactone) and TGF-β1 inhibitor successfully hinders over-proliferation of fibroblasts and wards off scarring. Gelatin nanofibers integrated with silicate particles promoted diabetic wound healing by regulating the release of silicon ions at the wound site [118]. It could be speculated that the nature of the dense and porous novel gelatin-based drug-eluting structures further proves the efficacy of gelatin biomaterial as an ideal drug carrier in wound healing applications.

Nonetheless, this study proves that it is less challenging to modify gelatin into different forms of drug carriers while preserving its natural properties, and particularly the ability to retain moisture at the wound site. These changes can ease the formulation of the microparticle or nanoparticle-based gelatin biomaterial which can be in the form of injection to deliver drugs at the specified location in the body. Along with it, the high water-absorption capacity of gelatin causes a rapid release profile, which prevents water-soluble medications from being released in a sustained and effective manner. Despite of the different sources of gelatin derivative, initial molecular weight, and degree of crosslinking can all affect water uptake to some extent, which contributes to the healing phase of the wound [119].

## 7. Conclusions and Future Prospective

In the healthcare industry, wound healing is the most critical area as any form of infection can result in severe complications. In this review, the authors compiled the existing knowledge of wound treatment and evaluated them critically to help researchers, clinicians and anyone interested in this field to understand the dynamic wound healing phenomenon, particularly focusing on the roles of the natural substances Col and gelatin. The authors introduced the principles of an ideal scaffold for wound management, paying particular attention to recent studies of native Col and gelatin as ideal biomaterials for wound healing. Despite their differing sources of derivative, both Col and gelatin are said to be ideal scaffolds and highly biocompatible to human cells, due to the integrin molecules. Their numerous properties (cost-efficient, biocompatible, swelling index) and the various manners of their introductions into the human system (electrospun fibers, hydrogels, sponge, films, etc.) contribute to their being favorable options for researchers. However, the uses of Col and gelatin for wound treatments are holistic approaches which are still at premature levels and lack large-scale clinical trials. The state-of-the-art technology nowadays is the use of biomaterials for 3D printing which imitates the extracellular matrix. The use of 3D-printed skin on a wound could provide an effective alternative in the field of wound care in the near future. Hence, our assessment of the current knowledge of the wound healing process, together with wound microbiology and the present status of wound dressings would be very helpful in the development of more effective wound dressings for individual patients as well as for the continued progress of the medical and pharmaceutical industries.

## Figures and Tables

**Table 1:** Differences between collagen and gelatin.

Property	Collagen	Gelatin
Origin	Animals/humans	Col from bones/skin
Precursor	Fibroblast	Col type I
Physical characteristics	Elastic, tough and versatile structural protein	Smooth and gel like substances
Number of Amino acids	Approximately 1050	Less than 20
Structure of peptide	Triple helix of polypeptide chain	Small peptides
Types	Fibril-forming and nonfibril forming	A and B
Aromatic radicals	Present	Absent
Solubility	NaCI solution/dilute acid	H_2_O
Mechanical strength	Poor	Poor
Antigenic response	Possible, in case of crosslinking/integration with antibacterial agent	Impossible, Because of its hydrophilicity nature.
Digestion	Difficult	Easy
Protease	Resistant	Susceptible
Gelling properties	No	Yes
In vitro degradation	Serine protease, pepsin-cleaving enzyme, gelatinease and collagenase	Collagenase
In vivo degradation	Endopeptidase	MMP-2 and MMP-9
Disease transmission	Xenozoonoses if the Col is impure	Not encountered
Usage	Burns, hemostasis, tissue defects, regeneration of nerves, wound dressings, augmentation of soft tissue, artificial dermis skin replacement	Adhesive of soft tissues, artificial skin, regeneration of nerves, wound dressings

**Table 2:** Different sources of collagen and gelatin.

Protein	Gelatin	Collagen
Sources	Mammalian, porcine, bovine, bone, fish skin (Labeo rohita), insects, cattle bones, hides, chicken, fins, sea urchin, jelly fish, bird feet (Encephalopat), camel skin, corn plant and seaweed.	Bovine, fish, porcine, sponges, jellyfish, star fish, prawn, sea urchin, squid, sea anemone, octopus, cuttlefish, bone (Thunnus obesus, skipjack tuna, japanese sea-bass, yellow sea bream, horse mackerel, Oreochromis niloticas), ovine tendon, buffalo skin, tobacco plant, yeast (Saccharomyces cerevisiae and Pichia pastoris), bacteria (Escherichia Coli) and insects.
Extraction methods	Alkaline hydrolysis, acid hydrolysis, thermal extraction and high pressure processing.	Chemical hydrolysis, enzymatic hydrolysis, acidic/ salting out, alkaline extraction and acidic extraction.
Composition	The amino acid composition differs depending on the source of derivative.	The extraction method and the source of Col influence the Col physicochemical properties.
Crosslinking	Physical, chemical and enzymatic	

**Table 4:** In vitro and in vivo studies of gelatin for skin wound healing.

Author	Objective	Study Design	Subject	Duration	Outcome	Conclusion
Lei et al., (2019) [93]	To study the effectiveness of self-healing gelatin	In vitro	L929 cellsand L02 cells	24 h and 72 h	‒ FTIR showed absorption band at 2 935 cm^−1^ and amide I band at 1640 cm^−1^. ‒ Healing effectiveness was at 50% at 40th and 90% at 60 min. ‒ Pore size ∼100 μm was seen. ‒ Equilibrium swelling ratio was ~1.28. ‒ The temperature at the maximum rate of weight loss 308.7 °C. ‒ Cell viability was >90% up to 72 h. ‒ Absence of cytotoxicity was recorded.	The fabricated self-healing gelatin has application prospects in biomedical fields.
Akhavan-Kharazian et al., (2019) [94]	To characterize gelatin as a potential agent for wound healing	In vitro	Human fibroblast cells	7 days	‒ FTIR showed absorption band at 3264 cm ^−1^ (amide A), 1675 cm ^−1^ (amide I), 1542 cm ^−1^ and (amide II). ‒ Addition of chitosan increased the swelling percentage. ‒ The elastic modulus, tensile strength and elongation at break were 1450 ± 31 MPa, 47.3 ± 2.1 3 MPa and 5 ± 0.2 %. ‒ WVTR result was 46.1 g/m^2^/h. ‒ A 16 mm diameter of zone of inhibition was recorded against Escherichia Coli. ‒ Good adhesion of fibroblast cells and viability was seen up to 7 days.	Gelatin has the potential to be integrated as a wound healing material.
Hsu et al., (2019) [95]	To study the efficacy of gelatin for wound healing in diabetic mice	In vivo	Male C57BL/6 J Narl mice	Up to 10 days	‒ Porous structure was in the range of 20 µm to 300 µm. ‒ FTIR showed absorption bands at 1650 cm^−1^ and 1545 cm^−1^ for amide I and amide II, respectively. ‒ The degradation rate increased proportionally to the amount of collagenase. ‒ Thick granulation tissues, increased re-epithelization and blood vessel formation were observed in the treated group.	Gelatin is capable of contributing to diabetic wound healing.
Nikpasand et al., (2019) [96]	To access the outcome of gelatin nanocomposite in wound healing	In vivo	50 male rats	Up to 21 days	‒ Wound contraction was seen over time starting from day 6. ‒ Re-epithelization and neovascularization was seen in the experimental group. ‒ High level of mononuclear cells polymorphonuclear cells and proliferation of fibroblast cell was seen. ‒ Hydroxyproline content was recorded at 97.88 ± 3.77 mg g^−1^ indicating a high level of Col deposition.	Gelatin nanocomposite accelerates wound healing.
Enrione et al., (2018) [103]	To study the efficacy of salmon gelatin in wound healing	In vivo	6 Orictholagus cuniculus rabbits	Not specified	‒ The pore size was 185.2 ± 27.1 µm ‒ Young modulus, stress at break and strain at break were 150.0 ±17.3 MPa, 316.8 ± 18.4 MPa and 2.48 ± 0.99%, respectively. ‒ DSC was recorded at 318.1 ± 0.5 K. ‒ Up to 93% of wound closure by week 4 was seen.	Salmon gelatin is a potential wound dressing material.
Garcia-Orue et al., (2019) [104]	To access the effectiveness of gelatin crosslinked with different agents for wound healing	In vitro and ex vivo assay	L-929 fibroblasts and redundant tissue from patients	Up to 8 days	‒ FTIR showed absorption bands at 1630 cm^−1^, 1530 cm^−1^, 1230 cm^−1^ for amide I, amide II and amide III. ‒ At 700% swelling equilibrium was reached. ‒ WVTR existed in the range of 773.7 ± 43.4 g/m^2^ day and 787.0 ± 50.9 g/m^2^. ‒ Absence of cytotoxicity confirmed through CCK-8 assay. ‒ >70% of cell viability was recorded on day 4 and day 8. ‒ Increase in cell proliferation markers, myofibroblast differentiation, cytokeratin 14 and 10 was seen.	Gelatin hydrofilm serves as a perfect biomaterial for wound dressing.
Zeng et al., (2015) [105]	To access the effectiveness of gelatin microcryogel for wound healing	In vitro and In vivo	human adipose-derived stem cells and nude mice	Up to 11 days	‒ Pore size was 400 µm width and 500 µm height. ‒ Swelling ratio was recorded at 23.49 ± 1.57%. ‒ Yong modulus was recorded at 8.25 ± 0.64 KPa. ‒ >65% of adherence of cells to the scaffold with 1.5 h. ‒ Gene expression study showed increase in vascular endothelial growth factor (VEGF), hepatocyte growth factor (HGF), basic fibroblast growth factor (bFGF) and platelet-derived growth factor (PDGF) at 48th hour.	Gelatin microcryogel supports wound healing.
Jang et al., (2017) [97]	To analyse the effectiveness of gelatin paste containing dermal powder for wound healing	In vitro and in vivo	Fibroblasts and Sprague Dawley rats	18th and 48th day	‒ On 18th day, 85% of wound contraction was seen in the gelatin group. ‒ A thick spinous layer and hyperkeratosis was seen on day 48. ‒ Low level of elastic fibers and blood vessel formation was seen in the controlled group. ‒ No significant immune response was seen. ‒ Leukocyte values were 7.28 ± 3.24 and 8.78 ± 2.71 for days 18 and 48, respectively.	Gelatin promotes full thickness wound healing.
Gomes et al., (2015) [106]	To evaluate the effectiveness of gelatin in skin wound healing	In vitro and in vivo	human fetal fibroblasts (HFFF2) and 18 Wistar rats	Up to week 4	‒ The porosity was recorded as 78 ± 10% with a lowest viscosity and highest conductivity. ‒ Gelatin scaffold was rigid with an elasticity result of 162 ± 96 MPa and brittle, ε = 9 ± 5%. ‒ FTIR showed 3280 cm^−1^, 1640 cm^−1^, 1530 cm^−1^ and 1240 cm^−1^ for amide A, amide I, amide II and amide III, respectively. ‒ Cell viability reduced to 67% on 2nd day of cell seeding. ‒ Continuous cell growth in gelatin scaffold was seen up to day 9. ‒ Cells were scattered in the gelatin scaffold which then improved in density and alignment by day 7. ‒ Rapid healing was recorded in in vivo study for gelatin group. ‒ Wound contraction, formation of new blood vessels and complete healing was achieved by week 4.	Gelatin scaffold supports full thickness wound healing.

**Table 5:** Current FDA approved skin healing products from native collagen.

Formulations	Product	Pharmaceutical Company	Indication	Contraindication	Benefits
Bovine derived Col type I	HyCol Collagen Powder	Sanara Med Tech Inc.	Chronic and acute wounds, partial and full-thickness wounds, all types of pressure injuries, venous stasis ulcers, arterial ulcers, diabetic ulcers, traumatic wounds, and 1st degree burns	Not recommended for those allergic to bovine derivative products.	Effective, versatile, easy to apply and compatible.
Bovine derived Col type I	Stimulen Collagen Powder	Southwest Technologies Inc.	Partial and full-thickness wounds, pressure ulcers, venous ulcers, diabetic ulcers, partial-thickness burns, acute wounds, abrasions, and traumatic wounds	Not recommended for those allergic to bovine derivative products.	One time application, prevents dehydration, ease of usage, provide moist microenvironment, non-cytotoxic nonirritating, exudates break down the Col powder.
Native equine type I Collagen	Biopad	L&R USA, Inc.	Pressure ulcers, venous insufficiency ulcers, diabetic ulcers, partial- and full-thickness wounds, surgical and traumatic wounds, draining wounds, lacerations, podiatric, and post-laser surgery	-	Rapidly heals hard to heal wounds.
Porcine derived Col type I	Biostep Collagen Matrix	Smith and Nephew, Inc.	Full and partial-thickness wound, pressure ulcers, diabetic ulcers, venous ulcers, abrasions, traumatic wounds, dehisced surgical wounds and 1st and 2nd degree burns	Not recommended for those allergic to porcine derivative products and 3rd degree burns.	Targets MMPs and optimizes moisture surface at the injury site. Ease of application.
Bovine derived Col type I	Collatek Collagen Gel	Human BioSciences, Inc	Abrasions, cuts, superficial injuries, severe sunburns, partial and full thickness wounds, venous stasis ulcers, 1st and 2nd degree burns, ulcers	Not recommended for those allergic to bovine derivative products.	Prevents dehydration, optimizes moisture microenvironment, fills cavity wounds, ease of application and cost effective.
Native Col	Cutimed Epiona	Essity	Full thickness skin loss	-	3D matrix that enhances skin regeneration, decreases enzymatic degradation, provides structural support, and enhances cell proliferation by creating ECM like structure.
Bovine derived Col type I	DermaCol 100	DermaRite Industries, LLC	Full and partial-thickness wounds, pressure ulcers, diabetic ulcers, venous ulcers, abrasions, traumatic wounds, dehisced surgical wounds and 1st and 2nd degree burns	Not recommended for those allergic to bovine derivative products and 3rd degree burns.	High absorbent capacity, optimize moist surface at the wound site, and aids hemostatic process.
Bovine derived Col type I	DermaCol 100 Sheet	DermaRite Industries, LLC	Moderately to heavily exuding wounds with minor bleeding	Not recommended for those allergic to bovine derivative products.	Optimizes moist surface at the wound site, absorb exudates, and aids hemostatic process.
Bovine derived Col	Gentell Collagen	Gentell	Burns, sores, blisters, scrapes and ulcers.	Not recommended for those allergic to bovine derivative products.	Regulates MMPs, aids in hard to heal wounds, and can be used at any stage of wound healing.
Bovine derived Col type I	Helix 3 Bioactive Collagen	Amerx Health Care Corp.	Burns, sores, blisters, ulcers, and exuding wounds	Not recommended for those allergic to bovine derivative products.	Optimizes moist surface at the wound site, absorbs exudates, effective in all wound healing phases, and available in many forms such as gels, powders, etc.
Bovine derived Col	Medifill II Collagen Particles	Human BioSciences, Inc.	Burns, sores, blisters, scrapes, and ulcers.	Not recommended for those allergic to bovine derivative products.	Absorbing capacity, on shelf biomaterial, optimizes moist microenvironment, allows gas exchange, and cost effective.
Bovine derived Col type I	PuraCol Plus MicroScaffold Collagen	Medline Industries, Inc.	Partial and full-thickness wounds, pressure ulcers, venous ulcers, diabetic ulcers, donor sites and other bleeding surface wounds, abrasions, trauma wounds, and 1st and 2nd degree burns.	Not recommended for those allergic to bovine derivative products.	Aids in chronic wounds, exudate absorbing capacity, optimizes moist microenvironment, and aids cell growth.
Bovine derived Col type I	PuraCol Ultra Powder	Medline Industries, Inc.	Partial and full-thickness wounds, pressure ulcers, venous ulcers, diabetic ulcers, donor sites and other bleeding surface wounds, abrasions, trauma wounds, and 1st and 2nd degree burns.	Not recommended for those allergic to bovine derivative products.	Bioabsorbent, optimizes moist microenvironment, and aids in regeneration of granulation tissue.
Undigested bovine derived Col	Simpurity Collagen Pad	Safe n’ Simple	Partial and full-thickness wounds, tunnel or undermined wounds or surgical wounds, low to moderately exuding chronic wounds including diabetic foot ulcers, venous leg ulcers and sores	Not recommended for those allergic to bovine derivative products.	High exudate absorption, retention provides moist wound healing environment, acts as hemostatic agent, and ease of application.
Bovine derived Col	SkinTemp II Collagen Sheets	Human BioSciences, Inc.	Burns, sores, blisters, scrapes and ulcers	Not recommended for those allergic to bovine derivative products.	Sustained adherence, hypoallergenic, non-pyrogenic, non-toxic, fluid control, impermeable to exogenous microorganisms, absorbs exudates, on shelf biomaterial, ease of application, and cost effective.
Animal derived Col	Stimulen Collagen Gel	Southwest Technologies, Inc.	Partial and full-thickness wounds, pressure ulcers, venous ulcers, diabetic ulcers, donor sites and other bleeding surface wounds, abrasions, trauma wounds, and 1st and 2nd degree burns.	Not recommended for those allergic to animal derivative products.	Prevents dehydration, optimizes moist microenvironment, fills cavity wounds, nontoxic, and nonirritating.
Type I Col	Triple Helix Collagen Dressing	MPM Medical, Inc.	1st and 2nd degree burns, full and partial-thickness wounds, pressure ulcer stages II-IV, diabetic vascular and venous insufficiency ulcers, and light to heavily exudating wounds.	-	Can be customized to any desired size.

## Data Availability

The data presented in this study are available on request from the corresponding author.
